# Late onset non-infectious pulmonary complications and pre-transplant pulmonary function as prognostic factors in allo-HCT using post-transplant cyclophosphamide

**DOI:** 10.3389/fimmu.2026.1875293

**Published:** 2026-08-04

**Authors:** Mónica Molina Martínez, Paloma Ortiz Cañas, María Suárez-Lledó, Gorka Pinedo, Ares Guardia, Laia Guardia, Cristina Moreno, Berta Solé, Paola Charry, Joan Cid, Miquel Lozano, Laura Rosiñol, Enric Carreras, Francesc Fernández-Avilés, Carmen Martínez, Carles Agustí, Montserrat Rovira, María Queralt Salas

**Affiliations:** 1 Hematopoietic Transplantation Unit and Hematology Department, Clinical Institute of Hematology and Oncology (ICAMS), Hospital Clínic de Barcelona, Barcelona, Spain; 2 Hematology Department, Hospital Universitario Clínico San Carlos, Madrid, Spain; 3 Institut d’Investigacions Biomèdiques August Pi i Sunyer (IDIBAPS), Barcelona, Spain; 4 Hematology Department, Hospital Universitario de Cruces, Bilbao, Spain; 5 Apheresis and Cellular Therapy Unit, Hemotherapy and Hemostasis Department, Institute of Cancer and Blood Diseases (ICAMS), Hospital Clínic de Barcelona, Barcelona, Spain; 6 University of Barcelona, Barcelona, Spain; 7 Fundació i Institut de Reserca Josep Carreras Contra la Leucèmia, Barcelona, Spain; 8 Pneumology Deparment, Hospital Clínic de Barcelona, Barcelona, Spain

**Keywords:** allo-HCT, NRM, outcomes, PTCY, pulmonary function test, toxicity

## Abstract

This study evaluates the incidence and clinical impact of late- onset non-infectious pulmonary complications (LONIPCs) in adults undergoing allo-HCT with post-transplant cyclophosphamide (PTCy). In addition, it assesses the prognostic value of pre-transplant pulmonary function tests (PFTs) for overall mortality in this population. A total of 317 consecutive adults transplanted between 2014 and 2024 were retrospectively included. LONIPC occurred in 10.6% of patients. The most frequent diagnoses were cryptogenic organizing pneumonia (COP, 4.1%), bronchiolitis obliterans syndrome (BOS, 5.9%), and pulmonary thromboembolism (0.6%). Risk factors for LONIPC included smoking history, pre-existing bronchial asthma, mismatched donors, and grade III–IV acute GVHD. The diagnosis of LONIPC was strongly associated with adverse outcomes in both univariate (OS: HR 9.37, *P* < 0.001; NRM: HR 4.19, *P* = 0.001) and multivariate analyses (OS: HR 8.07, *P* < 0.001; NRM: HR 3.19, *P* = 0.033). Based on pre-transplant PFTs and the HCT-CI scoring system, patients were classified into low- (24.3%), moderate- (35.0%), or high-risk groups (40.7%). High-risk patients had significantly poorer overall survival (OS) (3-year OS: 58.8% vs. 72.0% and 70.0%, P = 0.027) and higher non-relapse mortality (NRM) (3-year NRM: 19.0% vs. 7.9% and 15.1%) compared with those in the low- and intermediate-risk groups. No differences were observed regarding pre-transplant PFTs between patients developing LONIPC and those who did not. In conclusion, the incidence of LONIPC occurs in 10% of adults undergoing allo-HCT with PTCy. Its diagnosis, as well as the presence of significant pre-transplant PFT abnormalities, were associated with worse outcomes, underscoring their prognostic importance in the contemporary PTCy-based allo-HCT setting.

## Introduction

Allogeneic hematopoietic cell transplantation (allo-HCT) remains a potentially curative therapy for patients with high-risk hematologic malignancies (1, 2). In recent years, the introduction of post-transplant cyclophosphamide (PTCy) for graft-versus-host disease (GVHD) prophylaxis has profoundly reshaped allo-HCT practices. PTCy not only reduces the risk of GVHD while preserving the graft-versus-leukemia effect but has also become a widely adopted strategy across donor types worldwide (3–6). However, despite its efficacy in preventing GVHD, PTCy-based regimens are associated with delayed immune reconstitution and an increased risk of infectious complications (7–10).

Late-onset non-infectious pulmonary complications (LONIPC) such as cryptogenic organizing pneumonia (COP) or bronchiolitis obliterans syndrome (BOS) (11–13), represent a heterogeneous group of disorders that arise beyond three months after allo-HCT and carry substantial morbidity and mortality. These complications may affect all pulmonary structures—including bronchi, parenchyma, vasculature, and pleura—giving rise to diverse clinical syndromes. Although their pathogenesis is not fully understood, current evidence implicates factors such as chronic GVHD, conditioning regimens, and immune-mediated inflammation. Given the widespread adoption of PTCy and its distinct effects on immune reconstitution (7–10), a better characterization of post-transplant pulmonary complications in patients receiving PTCy-based prophylaxis is warranted.

PTCy-based prophylaxis has been systematically implemented at our institution as the standard GVHD prevention strategy across all donor types (14). This retrospective study, evaluates the incidence, risk factors, and clinical outcomes of LONIPC in adult patients who underwent their first allo-HCT with PTCy-based prophylaxis at our center. It also examines the predictive value of pre-transplant pulmonary function tests (PFTs) for identifying patients at increased risk for mortality.

## Methods

### Patient selection

This study includes all consecutive adult patients with hematological disorders who underwent allo-HCT at our institution from 2015 to 2023. All patients received PTCY-based prophylaxis and peripheral blood stem cell (PBSC) grafts. Data were collected retrospectively and last updated in January 2025. The study received approval from the Ethics Committee of Hospital Clínic de Barcelona and was conducted in accordance with the ethical principles of the Declaration of Helsinki.

### Pre-transplant pulmonary assessment and main definitions

Pre-transplant pulmonary assessments included pulmonary function tests (PFTs) and chest X-rays. Patients with significant abnormalities or a history of relevant pulmonary disease also underwent chest computed tomography (CT) scans prior to transplantation. Overall, based on PFT results, patients were stratified according to the pulmonary component of the Hematopoietic Cell Transplant – Comorbidity Index (HCT-CI). Specifically, patients with normal pulmonary function (FEV1 and DLCO ≥80% of predicted) were classified as low risk; those with mild impairment (FEV1 or DLCO 70–79%) were categorized as moderate risk; and patients with moderate to severe impairment (FEV1 or DLCO <70%) were assigned to the high-risk group.

The diagnosis, management and outcomes of the following LONIPC was investigated through retrospective chart review: cryptogenic organizing pneumonia (COP), bronchiolitis obliterans syndrome (BOS), idiopathic pneumonia syndrome (IPS), lung fibrosis, pulmonary thromboembolism (PTE) and pulmonary hypertension (PH) (11–13). During the study period, LONIPC complications diagnosed followed standard criteria and treated following established clinical guidelines, in close coordination with the hospital’s pulmonology team. This collaborative framework enabled a comprehensive, multidisciplinary strategy for patient assessment, therapeutic decision-making, and long-term follow-up evaluation. For the purposes of this study, only pulmonary complications occurring in patients in complete remission were included; cases diagnosed after disease relapse were excluded from the analysis.

### Allo-HCT methodology

The eligibility requirements for allo-HCT and the donor selection criteria are provided in the **Supplementary Material**. Patients older than 55 without significant comorbidities received myeloablative conditioning (MAC), which typically involved fludarabine (30 mg/m²/day IV for 4 days) combined with either busulfan (3.2 mg/kg/day IV for 4 days) or total body irradiation (TBI) at 12 Gy. In contrast, reduced-intensity conditioning (RIC) regimens included standard-dose fludarabine alongside lower doses of busulfan (3.2 mg/kg/day IV for 3 days) or TBI at 8 Gy. For haploidentical HCT (haplo-HCT), patients uniformly received 2 Gy TBI as part of their conditioning regimen.

All patients received PTCY-based prophylaxis, with specific protocols adjusted according to donor type. Patients receiving grafts from 10/10 HLA-matched sibling or unrelated, and 9/10 mismatched unrelated donors (MSD, MUD and MMUD) received double-based PTCy-based prophylaxis composed by PTCY at 50 mg/kg/day on days +3 and +4 and therapeutic tacrolimus (Tac) from day +5 to day +90. Patients undergoing haplo-HCT also received mycophenolate mofetil (MMF) from day +5 to day +30. In the absence of GVHD, tacrolimus was gradually tapered off and stopped by day +180.

Unmanipulated T cell–replete PBSC grafts were infused on day 0. Since November 2021, granulocyte colony-stimulating factor has been administered subcutaneously at 300 mcg daily from day +7 until the absolute neutrophil count surpasses 1.5 × 10^9/L. CMV reactivation was defined as the detection of CMV DNA in blood by quantitative PCR above the institutional threshold prompting clinical monitoring. CMV disease was defined according to established consensus criteria as evidence of CMV-associated end-organ involvement.

### Statistical analysis

The results of pre-transplant PFTs and the diagnosis of LONIPC after allo-HCT were considered the main exploratory variables. The cumulative incidence function of pulmonary complications was estimated using regression analysis and accounting for death and relapse as competing events. Mortality rate at 30 and 60 days was calculated from the date of LONIPC diagnosis for patients who died during subsequent follow-up.

Descriptive statistics were presented as counts and percentages. The Chi-square and Fisher tests and Mann-Whitney U test were used to assess statistically significant differences between subgroups. Overall survival (OS) and non-relapsed mortality (NRM) were the main outcome variables of interest. OS was analyzed using Kaplan-Meier survival and NRM with cumulative incidence regression analyses. Cumulative incidence of relapse (CIR) was also estimated and defined as the probability of disease recurrence over time, considering death without relapse as a competing event. Predictors of pulmonary events, OS and NRM were evaluated through univariate and multivariate regression models (UVA and MVA). The impact of LONIPC on outcomes was assessed using regression analyses. LONIPC, CMV reactivation, CMV disease, respiratory viral infections, and the diagnosis of GVHD were analyzed as time-dependent covariates when included in survival models. Patients contributed person-time to the unexposed category until the occurrence of the event and to the exposed category thereafter. All statistical tests were two-sided, with a significance level set at P <0.05. Statistical analyses were performed using EZR software (15).

## Results

### Baseline characteristics according to pre-transplant pulmonary function

A total of 317 patients were included in the study. As summarized in **Table 1**, the median age was 53 years (range: 18–75), and 194 (61.1%) were male. The most common underlying diagnoses were acute myeloid leukemia (AML; n=114, 35.9%), myelodysplastic syndrome (MDS; n=61, 19.2%), and acute lymphoblastic leukemia (ALL; n=58, 18.2%).

**Table 1:** Baseline information of the study cohort and according to pulmonary morbidity.

	All patients	Patients’ classification according to PFTs			
Baseline Characteristics	N=317	Low-Risk N=77	Mod-Risk N=111	High-Risk N=129	P value
Age median (range)	53 (18-75)	53 (18-74)	53 (18-72)	55 (18-75)	0.676
Older 64 years	53 (16.7)	12 (15.5)	19 (17.1)	22 (17.05)	0.954
Sex					
Male	194 (61.1)	54 (70.1)	64 (57.6)	76 (58.9)	0.178
Female	123 (38.8)	23 (29.8)	47 (42.3)	53 (41.08)	
Relevant comorbidities					
Active smoker	99 (31.2)	21 (27.2)	31 (27.9)	47 (36.4)	0.242
Asthma requiring treatment	12 (3.7)	2 (2.5)		4 (3.1)	0.554
COPD requiring treatment	13 (4.1)	3 (3.8)	6 (5.4) 1 (0.9)	9 (6.9)	0.061
Baseline Diagnosis					
Acute Myeloid Leukemia	114 (35.9)	20 (25.9)	40 (36.03)	54 (41.8)	0.005
Myelodisplastic Syndrome	61 (19.2)	15 (19.4)	26 (23.4)	20 (15.5)	
Myeloproliferative neoplasms	16 (5.04)	3 (3.8)	7 (6.3)	6 (4.6)	
Acute Lymphoblastic Leukemia	58 (18.2)	17 (22.07)	20 (18.01)	21 (16.2)	
Lymphoproliferative Disorders	41 (12.9)	13 (16.8)	10 (9)	18 (13.9)	
Multiple Myeloma	8 (2.52)	0	5 (4.5)	3 (2.3)	
Other*	19 (5.99)	9 (11.6)	3 (2.7)	7 (5.4)	
HCT-CI >2	161 (50.8)	6 (7.7)	16 (14.1)	109 (84.4)	<0.001
KPS <90%	74 (23.34)	13 (16.8)	28 (25.2)	33 (25.5)	0.305
Intensity					
Myeloablative	139 (43.8)	35 (45.4)	53 (47.7)	52 (40.3)	0.565
Reduced intensity	178 (56.1)	42 (54.5)	59 (53.1)	77 (59.6)	
Total Body Irradiation	124 (39.1)	34 (44.1)	41 (36.9)	49 (37.9)	0.574
Donor Type					
Matched sibling donor	46 (14.5)	7 (9.09)	20 (18)	19 (14.7)	0.661
10/10 Matched unrelated donor	113 (35.6)	30 (38.9)	34 (30.6)	49 (37.9)	
9/10 Mismatched unrelated donor	92 (29.02)	24 (31.1)		35 (27.1)	
Haploidentical	66 (20.8)	16 (20.7)	33 (29.7) 24 (21.6)	26 (20.1)	
Median Follow-up (months) (IQR)	29.9 (13.8-56.1)	27.7 (7.8–58.2)	35.7 (15.2–64.7)	27.7 (7.8–48.8)	0.193

*Other baseline diagnosis include: malignant hemopathies such as Blastic Plasmacytoid Dendritic Cell Neoplasm (BPDCN) or mycosis fungoides, and non-malignant hematological diseases such as aplastic anemia.

The p-values correspond to comparisons among patients stratified into low-, moderate-, and high-risk groups for post-transplant complications according to their pre-transplant pulmonary function tests (PFTs).

PFTs, pre-transplant pulmonary function tests; COPD, Chronic obstructive pulmonary disease requiring active treatment; HCT-CI, Hematopoietic Cell Transplantation – Comorbidity Index; KPS, Karnofsky Performance Status.

Based on pre-transplant PFTs, 77 (24.3%) patients were classified as low-risk (normal results), 111 (35.0%) as moderate-risk, and 129 (40.7%) as high-risk. Patients in the three PFT risk groups showed comparable median age, sex distribution, and pre-transplant KPS. However, the high-risk group had a higher proportion of patients diagnosed with AML (P = 0.005). Regarding pulmonary comorbidities, 99 (31.2%) patients were current or former smokers, 12 (3.7%) had a history of asthma, and 13 (4.1%) had chronic obstructive pulmonary disease (COPD). These factors were more frequent in the high-risk group, but the differences did not reach statistical significance for smoking (P = 0.242) or asthma (P = 0.554), though there was a trend toward significance for COPD (P = 0.061).

Overall, 139 (43.8%) patients received MAC, 46 patients (14.5%) received grafts from MSD, 113 (35.6%) from MUD, 92 (29.0%) from MMUD, and 66 (20.8%) from haploidentical donors, with no differences between groups (P = 0.661).

### Main post-transplant information

The main post-transplant results are summarized in **Table 2**. The median duration of transplant hospitalization was 29 days (IQR: 23-28). Median time to neutrophil and platelet engraftment was 18 (IQR 15-22) and 17 (IQR: 13-27) with no significant differences across pulmonary morbidity groups (P = 0.305 and P = 0.127). Graft failure occurred in 13 (4.1%) patients, with a cumulative incidence of 3.7% (95% CI: 1.8–5.9) by day +180, and no differences between groups (P = 0.743).

**Table 2:** Main post-transplant information.

	All patients	Patients’ classification according to PFTs			
Baseline Characteristics	N=317	Low-Risk N=77	Mod-Risk N=111	High-Risk N=129	P value
Median Duration HCT Hospitalization	29 (23-38)	28 (24-42)	29 (22-37)	30 (24-42)	0.579
Median days to neutrophil engraftment (IQR)	18 (15-22)	18 (16-23)	18 (15-21)	19 (16-23)	0.305
Median days to platelet engraftment (IQR)	17 (13-27)	15 (13-28)	16 (13-25)	20 (13-28)	0.127
Graft Failure, n (%)	13	2	5	6	0.771
Day +180 Cumulative Incidence of GF	3.7 (1.8-5.9)	1.3 (0.1-6.3)	4.5 (1.7-9.5)	2.9 (1.4-8.3)	0.743
Cumulative Incidence of Infectious Complications					
Day +180 CMV Reactivation	37.5 (32.2-42.9)	32.5 (22.3-43.0)	38.7 (29.7-47.7)	39.5 (31.1-49.9)	0.577
Day +180 CMV Disease	6.0 (3.7-9.0)	6.5 (2.4-13.5)	3.6 (1.2-8.3)	7.8 (4.0-13.2	0.347
2-Year Viral Respiratory Infection	27.3 (22.4-32.3)	32.3 (21.9-43.2)	28.8 (20.4-37.6)	23.3 (16.4-30.9)	0.108
2-Year Invasive Pulmonary Fungal Infection	11.4 (8.2-15.2)	5.2 (1.7-11.8)	13.6 (8.0-20.7)	13.2 (8.0-19.7)	0.093
Cumulative Incidence of GVHD					
Day +100 Grade II-IV aGVHD	20.8 (16.5-25.5)	18.2 (10.5-27.6)	17.1 (10.8-24.7)	25.6 (18.4-33.4)	0.282
Day +100 Grade III-IV aGVHD	6.6 (4.2-9.7)	5.2 (1.7-11.8)	5.4 (2.2-10.7)	8.5 (4.5-14.2)	0.398
2-Year Moderate/Severe cGVHD	9.2 (6.2-12.9)	5.6 (1.8-12.6)	11.4 (6.0-18.7)	9.5 (5.0-15.6)	0.502
Cumulative incidence of LONIPC					
2-year COP	4.1 (2.3-6.8)	5.3 (1.7-12.0)	2.7 (0.7-7.2)	4.7 (1.9-9.3)	0.638
2-year BOS	5.0 (2.9-8.5)	2.7 (0.5-8.5)	8.6 (4.2-15.0)	3.3 (1.1-7.6)	
2-year PTE	0.6 (0.1-2.1)	0	0	1.6 (0.3-5.0)	0.119
2-year pulmonary hypertension	0	0	0	0	0.232
Disease Relapse	90 (28.4)	23 (29.9)	31 (27.9)	36 (27.9)	0.947
Dead	105 (33.1)	21 (27.3)	31 (27.9)	53 (41.1)	0.044
Main Outcomes					
Overall Survival					
3-year	66.2 (60.4-71.4)	72.0 (59.2-81.4)	70.0 (60.5-78.8)	58.8 (49.5-67.0)	0.027
Relapse-Free Survival					
3-Year	56.6 (50.7-62.0)	58.7 (45.7-69.6)	57.4 (47.3-66.3)	53.4 (44.3-61.7)	0.428
Non-Relapse Mortality					
3-Year	14.8 (11.1-19.1)	7.9 (3.2-15.3)	15.1 (9.0-22.7)	19.0 (12.6-26.3)	0.189
Cumulative Incidence of Relapse					
3-Year	28.6 (23.6-33.8)	33.4 (22.1-45.2	27.4 (19.2-36.3	27.6 (20.1-35.6)	0.901

*Confirmed cases of lung cGVHD.

The p-values correspond to comparisons among patients stratified into low-, moderate-, and high-risk groups for post-transplant complications according to their pre-transplant pulmonary function tests (PFTs).

The cumulative incidence of post-transplant complications has been calculated considering dead and relapse as competing events.

PFT, pre-transplant pulmonary function tests; CMV, Cytomegalovirus; GVHD, Graft-versus-Host Disease; COP, cryptogenic organizing pneumonia; BOS, bronchiolitis obliterans syndrome; PTE, Pulmonary Thromboembolism.

Infectious complications were common in the early post-transplant period. As shown in **Table 2**, by day +180, CMV reactivation occurred in 37.5% (95% CI: 32.2–42.9) of patients, while CMV disease was diagnosed in 6.0% (95% CI: 3.7–9.0), with no significant variation between pulmonary morbidity groups (P = 0.577 and P = 0.347, respectively). At two years, 27.3% (95% CI: 22.4–32.3) of patients had at least one viral respiratory infection, and 11.4% (95% CI: 8.2–15.2) had probable or proven pulmonary fungal infections. Although these infections were numerically more frequent in higher-risk pulmonary groups, the differences did not reach statistical significance (P = 0.108 and P = 0.093, respectively).

The cumulative incidence of grade II–IV and III–IV aGVHD by day +100 was 20.8% (95% CI: 16.5–25.5) and 6.6% (95% CI: 4.2–9.7), respectively, without significant differences among groups (P = 0.282 and P = 0.398). Lastly, at 2 years, moderate-severe cGVHD was 9.2% (95% CI: 6.2–12.9), with no differences between groups (P = 0.282).

### Clinical outcomes according to pulmonary morbidity

**Figure 1:**
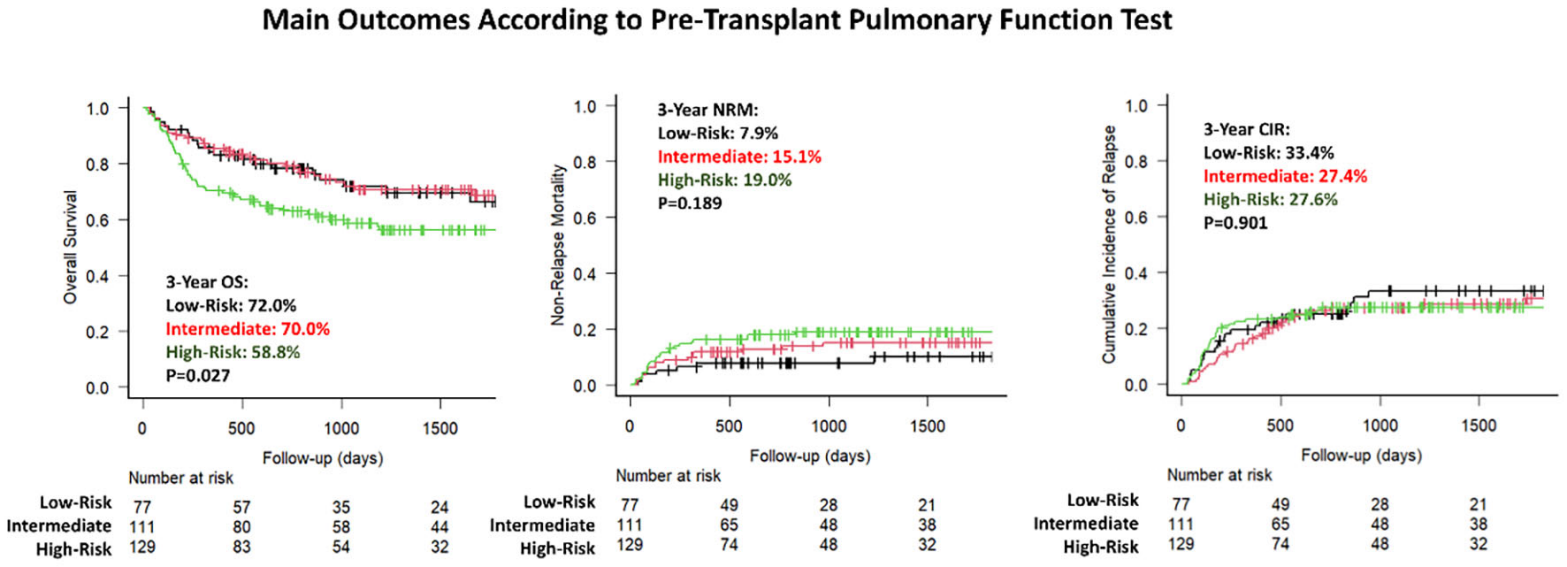
Post-transplant pulmonary complications and main outcomes according to pre-transplant pulmonary function test.

With a median follow-up of 30 months (IQR: 14-56), 90 (28.3%) patients relapsed and 105 (33.1%) died. As shown in **Table 2**; **Figure 1**, relapse and mortality outcomes were examined in relation to PFT results. Relapse rates were comparable between groups (3-year CIR rates of 33.4%, 27.4%, and 27.6%, respectively) among patients classified into low, moderate and high-risk pulmonary groups (P = 0.901). Conversely, rates of OS were lower in high-risk patients than in low-risk and moderate-risk patients (3-year: 58.8% vs. 72.0% and 70.0%, respectively, P = 0.027), especially secondary to a non-significant trend to higher NRM (3-year: 19.0% vs. 7.9% and 15.1%, respectively, P = 0.189).

### Incidence of LONIPC

As shown in **Table 3**; **Figure 1**, the overall incidence of LONIPC was low, with COP (n=13, 5.9%), BOS (n=19, 5.9%), and PTE (n=2, 0.6%) being the most prevalent entities. In contrast, no cases of IPS, or PH were documented during the study period.

**Table 3:** LONIPC after Allo-HCT with PTCY.

Baseline Characteristics	COP N=13	BOS N=19	PTE N=2
Age, years, median (range)	54 (21-68)	50 (18-70)	30 (18-42)
Sex			
Male	10 (76.9)	14 (73.7)	0
Female	3 (23.1)	5 (26.3)	2 (100)
Relevant comorbidities			
Active smoker	8 (61.5)	5 (26.3)	0
Asthma requiring treatment	1 (7.7)	4 (21.0)	0
COPD requiring treatment	2 (15.4)	1 (5.3)	0
Pre-Tx Pulmonary Function Test			
Low-Risk	4 (30.8)	3 (15.8)	0
Moderate-Risk	3 (23.1)	9 (47.4)	0
High-Risk	6 (46.2)	7 (36.8)	2 (100)
Diagnosis			
Acute Myeloid Leukemia	6 (46.2)	6 (31.6)	0
Myelodisplastic Syndrome	2 (15.4)	1 (5.3)	0
Myeloproliferative neoplasms	0	2 (10.2)	0
Acute Lymphoblastic Leukemia	3 (23.1)	5 (26.3)	2 (100)
Lymphoproliferative Disorders	2 (15.4)	3 (15.8)	0
Multiple Myeloma	0	2 (10.5)	0
Other			
KPS <90%	5 (38.5)	1 (5.3)	0
Intensity			
Myeloablative	7 (53.8)	7 (36.8)	2 (100)
Reduced intensity	6 (46.2)	12 (63.2)	0
Conditioning Regimen (Extended)			
Flu/TBI12	4 (30.8)	3 (15.8)	2 (100)
Flu/TBI8	1 (7.7)	0	0
Flu/Bu4 (+/- TBI2)	3 (23.1)	3 (15.8)	0
Flu/Bu3 (+/- TBI2)	4 (30.8)	12 (63.2)	0
Secuencial	1 (7.7)	0	0
Donor Type			
10/10 HLA-Matched Donors	3 (23.1)	5 (26.3)	1 (50.0)
9/10 MMUD / Haploidentical	10 (76.9)	14 (73.7)	1 (50.0)
Median Days to Diagnosis, IQR	6.3 (5.2–9.4)	16.0 (7.9–20.6)	1.7 (0.4–1.7)
Previous Infectious Complications (Dx in a range of 180 days)			
Pulmonary Fungal Infection	2 (15.4)	2 (10.5)	0
Respiratory Viral Infection*	3 (23.1)	1 (Rhinovirus)	0
Prior Diagnosis of Grade 3-4 aGVHD	4 (30.8)	2 (10.5)	1(50.0)
Prior Diagnosis of Mod/Sev cGVHD **	1 (7.7)	N/A	0
Concomitant Complications			
Pulmonary Concomitant Infection	0	0	0
Active aGVHD	0	0	0
Any grade of cGVHD	0	17 (89.4)	0
Mod/Sev cGVHD		9 (47.4)	
ICU Admission	2 (15.4)	0	0
Day +30 Mortality Rate	2 (15.4)	0	0
Day +60 Mortality Rate	2 (15.4)	0	0
Death during Follow Up	4 (30.8)	1 (5.3)	1 (50.0)
Disease Response at Last Follow-Up (Alive Patients)	N=9	N=18	N=1
Complete Response	9 (100)	2 (11.1)	1 (100)
Ongoing treatment	0	16 (88.9)	0

*Coronavirus (x1) and VRS (X2); **Affecting different organs other than lungs.

COPD, Chronic obstructive pulmonary disease requiring active treatment; KPS, Karnofsky Performance Status; GVHD, Graft-versus-Host Disease; Flu, fludarabine; TBI, total body irradiation; Bu, busulfan; MMUD, mismatched unrelated donor; ICU, Intensive Care Unit; FAM therapy, fluticasone–azithromycin–montelukast therapy; LMWH, Low molecular weight heparin.

COP was diagnosed in 13 patients at a median of 6 months after transplantation (IQR: 5–9), and resulting in a 2-year cumulative incidence of 4.1% (95% CI: 2.3–6.8%). At diagnosis, two patients (15.3%) required admission to the intensive care unit (ICU). At COP diagnosis, no patient exhibited concomitant acute or chronic GVHD involving other organs. All patients were treated with first-line systemic corticosteroids, resulting in an initial clinical improvement. Corticosteroid tapering was performed gradually with 10 (84.6%) patients achieving a complete response and 2 (15.3%) patients requiring a second-line therapy due to persistent or recurrent symptoms. Although no deaths were directly attributed to COP, 4 (30.7%) patients died during follow-up, with day +30 and +60 mortality rates of 15.4% each.

BOS was diagnosed in 19 patients at a median of 16 months after transplantation (IQR: 8–20), and with a 2-year cumulative incidence of 5.0% (95% CI: 2.9–8.5%). At diagnosis, 9 (47.3%) patients exhibited concurrent moderate or severe cGVHD involving other organs. FAM therapy was initiated in 18 patients. Systemic corticosteroids were administered to 5 patients, and systemic immunosuppression was introduced in 16 cases either during follow-up or concomitantly. No deaths were directly attributed to BOS; however, 16 (84.2%) patients still require ongoing treatment. In addition, one (5.3%) patient died during follow-up due to respiratory viral and fungal infections, and no mortality events were observed at day +30 and +60 after BOS diagnosis.

Finally, 2 (0.6%) cases of PTE were recorded, diagnosed at a median of 52 days post-transplant (IQR: 13–52). No cases of PH, lung fibrosis or IPS were observed during the study period.

### Risk factors for the most prevalent LONIPC: COP and BOS

Risk factors for the most frequent LONIPC documented during the study period were investigated using univariate regression analyses. As described in **Table 4**, a history of smoking (HR 3.63, P = 0.023), pre-existing asthma (HR 4.60, P = 0.012), and a prior diagnosis of grade III–IV aGVHD (HR 4.32, P = 0.022) were identified as risk factors for being diagnosed with COP. In contrast, BOS was significantly associated with a prior history of asthma (HR 6.24, P<0.001), transplantation from MMUD or haploidentical donors (HR 2.52, P = 0.043), and preceding viral respiratory infection (HR 2.52, P = 0.043).

**Table 4:** Risk factors for COP and BOS. Univariate regression analysis.

Univariate Analysis	Risk Factors for COP HR (95% CI)	P value	Risk Factors for BOS HR (95% CI)	P value
**Age median (range)**	0.99 (0.96-1.03)	0.88	0.98 (0.95-1.01)	0.28
Sex				
Female (vs. Male)	0.46 (0.12-1.69)	0.25	0.54 (0.19-1.48)	0.23
**Relevant comorbidities** History of smoker (vs. No) Asthma on treatment (vs. No) COPD on treatment (vs. No)	3.63 (1.19-11.05) 4.60 (1.40-15.13) 0	0.023 0.012 -	0.77 (0.27-2.16) 6.24 (2.16-18.05) 0	0.63 <0.001 -
**Pre-Transplant Pulmonary Function Test** Moderate-Risk (vs. Low) High-Risk (vs. Low)	0.51 (0.11-2.26) 0.90 (0.25-3.19)	0.23 0.88	2.04 (0.55-7.58) 1.33 (0.34-5.13)	0.29 0.68
**Pre-Transplant Pulmonary Function Test** High-Risk (vs. Low and Moderate-Risk)	0.72 (0.22-2.32)	0.59	2.48 (0.56-10.89)	0.23
**HCT-CI ** >1 (vs. 0-2)	0.61 (0.19-1.87)	0.39	0.43 (0.16-1.12)	0.08
**KPS** <90% (vs. KPS 90-100%)	2.08 (0.68-6.36)	0.2	0.58 (0.17-2.01)	0.39
**Intensity** Reduced intensity (vs. Myeloablative) **TBI use** High Dose (>=8 Gys) (vs. No TBI)	0.66 (0.22-1.96) 1.67 (0.51-5.39)	0.46 0.39	1.33 (0.52-3.35) 0.72 (0.21-2.46)	0.54 0.61
**Donor Type** MMUD / Haplo (vs. HLA-matched)	3.37 (0.92-12.25)	0.065	2.78 (1.01-7.67)	0.046
**Diagnosis of CMV React** Time-Dependent	1.08 (0.35-3.20)	0.885	1.39 (0.57-3.42)	0.461
**Diagnosis of CMV Disease** Time-Dependent	2.87 (0.66-12.53)	0.159	2.39 (0.72-7.97)	0.15
**Diagnosis of Respiratory Viral Infection** Time-Dependent	2.14 (0.62-7.35)	0.226	2.52 (1.02-6.22)	0.043
**Diagnosis of Grades II-IV aGVHD** Time-Dependent	1.61 (0.50-5.11)	0.418	1.96 (0.78-4.95)	0.150
**Diagnosis of Grades III-IV aGVHD** Time-Dependent	4.32 (1.23-15.17)	0.022	1.63 (0.37-7.18)	0.513
**Diagnosis of Mod-Sev cGVHD** Time-Dependent	2.42 (0.31-18.67)	0.394	N/A	

Variables labeled as time-dependent were modeled as time-varying covariates in the univariate and multivariate analysis.

COP, cryptogenic organizing pneumonia; BOS, bronchiolitis obliterans syndrome; PTE, Pulmonary Thromboembolism; COPD, chronic obstructive pulmonary disease; HCT-CI, hematopoietic cell transplantation–comorbidity index; KPS, Karnofsky performance status; TBI, Total Body Irradiation; MMUD, mismatched unrelated donor; Haplo, haploidentical donor; GVHD, Graft-versus-Host Disease.

### Impact of pulmonary morbidity and complications on outcomes

The impact of pre-transplant pulmonary impairment and the LONIPC diagnosis on OS and NRM were evaluated (**Table 5**; **Supplementary Table 1**, UVA). The UVA showed that patients classified as high-risk based on pre-transplant PFTs had a significantly increased risk of overall mortality (HR 1.68, P = 0.044) and a non-significant trend toward higher NRM (HR 2.13, P = 0.077). Furthermore, the diagnosis of LONIPC post-allo-HCT was associated with inferior OS (HR 9.37, P<0.001), primarily due to increased NRM (HR 4.19, P = 0.001).

**Table 5:** Risk factors for OS and NRM.

Univariate analysis	Risk Factors for OS HR (95% CI)	P value	Risk Factors for NRM HR (95% CI)	P value
**Pre-Transplant Pulmonary Function Test** Moderate-Risk (vs. Low) High-Risk (vs. Low)	0.99 (0.57-1.73) 1.68 (1.01-2.79)	0.998 0.044	1.61 (0.66-3.90) 2.13 (0.92-4.92)	0.290 0.077
**Post-Transplant Pulmonary Complications** All. Time-Dependent	7.38 (6.11-14.41)	<0.001	4.19 (1.66-10.54)	0.001
MVA				
**Pre-Transplant Pulmonary Function Test** High-Risk (vs. Others)	8.07 (5.17-12.60)	<0.001	3.19 (1.09-9.32)	0.033
**Diagnosis of LONIPC after allo-HCT** All*. Time-Dependent	1.62 (1.09-2.42)	0.016	1.60 (0.88-2.87)	0.115
**Age** Continuous variable	1.02 (1.01-1.04)	0.005	1.04 (1.01-1.07)	0.001
**Disease Risk Index** High/Very-High (vs. Low/Int)	2.18 (1.44-3.32)	<0.001	1.36 (0.62-2.95)	0.436
**KPS** <90% (vs. KPS 90-100%)	1.94 (1.28-2.93)	0.001	3.17 (1.74-5.78)	<0.001
**Intensity** Reduced intensity (vs. Myeloablative)	1.14 (0.66-1.96)	0.623	0.69 (0.31-1.50)	0.356
**Donor Type** MMUD/Haplo (vs. HLA-Matched)	1.57 (1.04-2.38)	0.003	2.52 (1.29-4.93)	0.006
**Diagnosis of Grade III-IV aGVHD** Time-Dependent	3.80 (2.11-6.85)	<0.001	6.06 (2.71-13.53)	<0.001

*All diagnosis of LONIPC after allo-HCT include COP (cryptogenic organizing pneumonia), BOS (bronchiolitis obliterans syndrome) and PTE (pulmonary thromboembolism).

Variables labeled as time-dependent were modeled as time-varying covariates in the univariate and multivariate analysis.

KPS, Karnofsky performance status; TBI, Total Body Irradiation; MMUD, mismatched unrelated donor; Haplo, haploidentical donor; GVHD, Graft-versus-Host Disease.

These negative associations were confirmed in the MVA, which adjusted for other variables considered clinically relevant for the transplant success. Pre-transplant moderate-to-severe pulmonary impairment remained significantly associated with worse OS (HR 1.62, P = 0.016). Moreover, LONIPC diagnosis was confirmed to be an independent predictor of both inferior OS (HR 8.07, P<0.001) and higher NRM (HR 3.19, P = 0.033).

## Discussion

The present study shows that LONIPC—particularly COP and BOS-, occurs in approximately 10% of patients undergoing contemporary PTCy-based allo-HCT. Although the overall incidence is low, the development of LONIPC is associated with worse overall survival and increased non-relapse mortality. Pre-transplant PFTs impairment does not appear to predict the subsequent development of LONIPC; however, severe pre-transplant abnormalities are themselves linked to poorer overall survival.

PTCy has markedly transformed allo-HCT practice, reducing GVHD rates across donor types while preserving the graft-versus-leukemia effect (16). Its widespread adoption has been consistently associated with lower rates of both acute and chronic GVHD compared with classical prophylactic strategies (3–6). However, the intensified immunosuppression associated with PTCy has also been linked to delayed immune reconstitution, increased susceptibility to viral and fungal infections, and the emergence of a potentially distinct spectrum of immune-mediated complications in patients undergoing allo-HCT (7–10). Given this evolving clinical context, we aimed to re-evaluate both the incidence and clinical relevance of LONIPCs in the setting of PTCy-based allo-HCT.

LONIPC occurred in 10.6% of patients in our cohort, a rate that appears comparable to the incidences reported in historical allo-HCT series using conventional GVHD prophylaxis strategies, where estimates generally range between 10% and 20% depending on the definitions used and duration of follow-up (21–23). The most frequents diagnoses were bronchiolitis obliterans syndrome (BOS, 5.9%), cryptogenic organizing pneumonia (COP, 4.1%), and pulmonary thromboembolism (0.6%). Although direct comparisons between studies should be interpreted cautiously due to differences in patient populations, donor sources, conditioning regimens, and diagnostic criteria, the incidence of BOS and COP observed in our cohort falls within the ranges reported in previous allo-HCT studies.

In our population, BOS was diagnosed at a median of 16 months post-transplantation. BOS is characterized by progressive airflow obstruction resulting from fibrotic narrowing of small airways, typically associated with cGVHD. Nearly 90% of BOS patients had concurrent cGVHD, with half of them presenting with moderate to severe forms. Most patients received FAM therapy (fluticasone, azithromycin, and montelukast), and the majority required additional prolonged periods of systemic corticosteroids or other immunosuppressive agents during follow-up (17, 18).

Our study identified several predisposing factors for BOS, including prior asthma, alternative donor types (MMUD or haplo), and preceding viral respiratory infections. This is consistent with existing literature suggesting that chronic immune dysregulation and delayed immune recovery —often exacerbated by recurrent infectious insults—play a central role in the pathogenesis of BOS (19, 20). Although mortality was not directly attributable to BOS, its diagnosis resulted in prolonged morbidity and a higher need for immunosuppressive therapy, generating transplant morbidity and ending into a lower OS.

COP is an inflammatory pulmonary complication characterized by the deposition of granulation tissue obstructing bronchioles and alveolar ducts, resulting in respiratory symptoms and characteristic radiographic abnormalities. In the present series, the 2-year cumulative incidence of COP was 4.1%, consistent with previously reported rates ranging from 2% to 6% in allo-HCT cohorts. COP typically developed at a median of six months post-transplant (21–23). All COP cases received corticosteroids with initial improvement; however, retreatment after relapse and the inclusion of a second-line treatment was necessary in all cases (24, 25).

As a rule, all complications were promptly identified and managed in accordance with established institutional and international clinical guidelines, in close coordination with the hospital’s pulmonology team. This collaborative approach ensures comprehensive, multidisciplinary evaluation, timely therapeutic intervention, and structured long-term follow-up. While direct mortality due to LONIPC was rare, their occurrence was independently associated with significantly worse OS and increased NRM. These findings suggest that the burden of LONIPC goes beyond immediate respiratory compromise and may reflect a chronic organ injury that negatively affects patient outcomes. Moreover, even in the absence of direct pulmonary mortality, these complications may contribute to prologued immunosuppressive treatment, infections, and progressive functional decline, thus indirectly influencing survival (21–23).

Interestingly, pre-transplant PFT impairment, observed in nearly 41% of the cohort, did not predict the subsequent development of LONIPC. One possible explanation is that LONIPCs are primarily driven by post-transplant events, including immune dysregulation, alloimmune injury, infections, and chronic GVHD, rather than by pre-existing pulmonary dysfunction. In contrast, impaired baseline pulmonary function may reflect reduced physiological reserve, increased frailty, and a greater burden of comorbidities, thereby contributing to inferior overall survival independently of LONIPC development. Consistent with previous reports, high-risk PFTs were associated with poorer OS, reinforcing the value of pre-transplant PFTs as a broad risk stratification tool, as reflected in their incorporation into the HCT-CI, a widely validated scale for assessing pre-transplant comorbidities and predicting outcomes before allo-HCT (17–20).

The retrospective design represents the principal limitation of the present study. Moreover, the lack of a contemporaneous non-PTCy comparator prevents direct evaluation of the impact of PTCy on LONIPC. Given the progressive implementation of PTCy as the institutional standard, historical comparisons would have been difficult to interpret because of temporal differences in patient management. Second, the diagnosis of LONIPC was mostly based on clinical and radiographic findings without histological confirmation, which is common in clinical practice, but reduces diagnostic certainty. In addition, although the study included a large and homogeneously treated cohort, it reflects the experience of a single institution, potentially limiting generalizability.

Despite these limitations, our study provides valuable insights into the pulmonary complications occurring after PTCy-based allo-HCT in a real-world setting. It underscores that although the prevalence of LONIPC is low in this transplant setting, these complications remain clinically significant, as they increase morbidity and negatively impact overall outcomes (21–23). Our findings support the need for continued surveillance and early recognition strategies, as well as further research aimed at developing effective preventive and therapeutic interventions.

In conclusion, although the incidence of LONIPC remains relatively low in the contemporary PTCy-based allo-HCT setting, these complications carry substantial clinical relevance, as they are associated with worse long-term outcomes and increased non-relapse mortality. The absence of a clear predictive relationship between pre-transplant PFT abnormalities and the development of LONIPC highlights the complexity of their pathogenesis, while the association between severe pre-transplant impairment and poorer overall survival reinforces the value of comprehensive pulmonary assessment before transplantation. Collectively, these findings underscore the importance of vigilant post-transplant surveillance, timely recognition of pulmonary complications, and the need for prospective studies aimed at elucidating underlying mechanisms and developing more effective preventive and therapeutic strategies.

## Data Availability

The raw data supporting the conclusions of this article will be made available by the authors, without undue reservation.
