# Supplementary material for: Late onset non-infectious pulmonary complications and pre-transplant pulmonary function as prognostic factors in allo-HCT using post-transplant cyclophosphamide
Source: Front Immunol. 2026 Aug 4;17:1875293. doi: 10.3389/fimmu.2026.1875293 (PMC13481226; doi:10.3389/fimmu.2026.1875293)
Supplement: Supplementary file 1 [file DataSheet1.pdf]

SUPPLEMENTARY MATERIAL

Supplementary Table 1. Risk Factors for OS and NRM. Univariate Analysis

Univariate Analysis	Risk Factors for OS HR (95% CI)	P value	Risk Factors for NRM HR (95% CI)	P value
**Age**				
Older than 65 years (vs. younger)	1.02 (1.01-1.04)	<0.001	1.02 (1.01- 1.04)	0.017
**Sex**				
Female (vs. Male)	1.14 (0.77-1.68)	0.508	1.08 (0.60-1.94)	0.78
**Relevant comorbidities**				
History of smoker (vs. No)	1.16 (0.77-1.74)	0.495	1.25 (0.69-2.27)	0.45
Asthma (vs. No)	1.15 (0.50-2.63)	0.738	0	-
COPD (vs. No)	1.34 (0.65-2.76)	0.421	0.31 (0.04-2.23)	0.25
**Disease Risk Index**				
High/Very-High (vs. Low / Int)	3.68 (1.795-4.01)	<0.001	1.82 (1.01-3.31)	0.047
**HCT-CI**				
>2 (vs. 0-2)	1.55 (1.05-2.30)	0.026	1.48 (0.83-2.64)	0.18
**KPS**				
<90% (vs. KPS 90-100%)	2.23 (1.49-3.32)	<0.001	3.25 (1.83- 5.76)	<0.001
**Intensity**				
Reduced intensity (vs. Myeloablative)	1.80 (1.19-2.71)	0.004	1.18 (0.66-2.10)	0.57
**Donor Type**				
MMUD / Haplo (vs. MSD / MUD)	1.11 (0.75-1.63)	0.587	2.23 (1.21-4.11)	0.01
**Diagnosis of Grades II-IV Agvhd**				
Time-Dependent	1.37 (0.88-2.13)	0.155	2.82 (1.56-5.09)	<0.001
**Diagnosis of Grades III-IV Agvhd**				
Time-Dependent	4.04 (2.32-7.01)	<0.001	8.49 (4.56-15.78)	<0.001

Legend: Variables labeled as time-dependent were modeled as time-varying covariates in the univariate and multivariate analysis.

HCT-CI: hematopoietic cell transplantation–comorbidity index; KPS: Karnofsky performance status; TBI: Total Body Irradiation; MMUD: mismatched unrelated donor; Haplo: haploidentical donor; GVHD: Graft-versus-Host Disease.
